# Timing and impact of percutaneous endoscopic gastrostomy insertion in patients with amyotrophic lateral sclerosis: a comprehensive analysis

**DOI:** 10.1038/s41598-024-56752-5

**Published:** 2024-03-26

**Authors:** Bugyeong Son, Jisu Lee, Soorack Ryu, Yongsoon Park, Seung Hyun Kim

**Affiliations:** 1 Cell Therapy Center, Hanyang University Seoul Hospital, 222-1 Wangsimni-ro, Seongdong-gu, Seoul, 04763 Korea; 2 Department of Food and Nutrition, Hanyang University, 222 Wangsimni-ro, Seongdong-gu, Seoul, 04763 Korea; 3 Biostatistical Consulting and Research Lab, Medical Research Collaborating Center, Hanyang University, Seoul, Korea; 4 Department of Neurology, Hanyang University Seoul Hospital, 222-1 Wangsimni-ro, Seongdong-gu, Seoul, 04763 Korea

**Keywords:** Motor neuron disease, Health care, Nutrition

## Abstract

Dysphagia is common in amyotrophic lateral sclerosis (ALS) patients, often requiring percutaneous endoscopic gastrostomy (PEG) for enteral nutrition. We retrospectively analyzed data from 188 Korean patients with ALS who underwent PEG tube insertion at five-time points: symptom onset (t_1_), diagnosis (t_2_), recommended time for gastrostomy (t_3_), PEG insertion (t_4_), and one-year post-insertion (t_5_). The recommended time point for gastrostomy (T_-rec_ for gastrostomy) was defined as the earlier time point between a weight loss of more than 10% and advanced dysphagia indicated by the ALSFRS-R swallowing subscore of 2 or less. The T_-rec_ for gastrostomy was reached at 22 months after symptom onset, followed by PEG insertion at 30 months, resulting in an 8-month delay. During the delay, the ALSFRS-R declined most rapidly at 1.7 points/month, compared to 0.8 points/month from symptom onset to diagnosis, 0.7 points/month from diagnosis to T_-rec_ for gastrostomy, and 0.6 points/month after the PEG insertion. It is crucial to discuss PEG insertion before significant weight loss or severe dysphagia occurs and minimize the delay between the recommended time for gastrostomy and the actual PEG insertion. A stratified and individualized multidisciplinary team approach with careful symptom monitoring and proactive management plans, including early PEG insertion, should be prioritized to improve patient outcomes.

## Introduction

Amyotrophic lateral sclerosis (ALS) is a fatal neurodegenerative disease characterized by the progressive loss of motor neurons in the brain, brainstem, and spinal cord^1^. ALS generally presents as progressive voluntary muscle weakness, including the bulbar segment, typically resulting in respiratory failure and ultimately death within 2–4 years of diagnosis^2,3^. Despite heterogeneous clinical and genetic manifestations, patients with advanced-stage or bulbar-onset ALS inevitably suffer from swallowing difficulties, which can lead to critical nutritional challenges and life-threatening complications^4^. Furthermore, nutritional status is an independent predictor of disease progression and survival in ALS^5–9^. Therefore, optimal supportive management of dysphagia is considered an essential aspect of palliative care for patients with ALS^10,11^.

Noninvasive interventions, such as dietary modification of food texture, industrialized thicker liquids, and rehabilitation, can be the initial management approaches for patients with dysphagia^5,10,12,13^. However, for patients with severe dysphagia and malnutrition, various enteral feeding options are recommended, including L-tube insertion, gastrostomy, and jejunostomy^14^. Percutaneous endoscopic gastrostomy (PEG) is a commonly recommended enteral nutrition procedure for dysphagia^12^, which involves the insertion of a feeding tube through the abdominal wall directly into the stomach using a gastrofibroscope^6,9,15^.

Early PEG insertion guidelines are recommended to prevent weight loss and reduce the risk of complications associated with PEG procedures, including laryngeal spasms, local infection, gastric hemorrhage, technical difficulties leading to failed PEG placement, and respiratory arrest resulting in death^12,16^. Furthermore, clinicians recommend earlier PEG tube insertion in all patients with ALS with progressive dysphagia^17^. Despite these recommendations, patients and their families often express hesitancy regarding PEG tube insertion when oral intake is still possible^18^. Consequently, some patients inevitably experience aspiration pneumonia or malnutrition^10,19^.

Therefore, a comprehensive analysis of practical data regarding the timing of PEG tube insertion and its impact on prognosis in a large cohort of patients with ALS could provide valuable insights for clinical decision-making. The present study aimed to analyze the clinical data of patients with ALS who underwent PEG tube insertion and compare the practice with ideal recommendations. By examining real-world clinical practice, we aimed to gain a better understanding of the optimal timing for PEG tube insertion and its impact on patient outcomes.

## Methods

### Study design and participants

**Figure 1:**
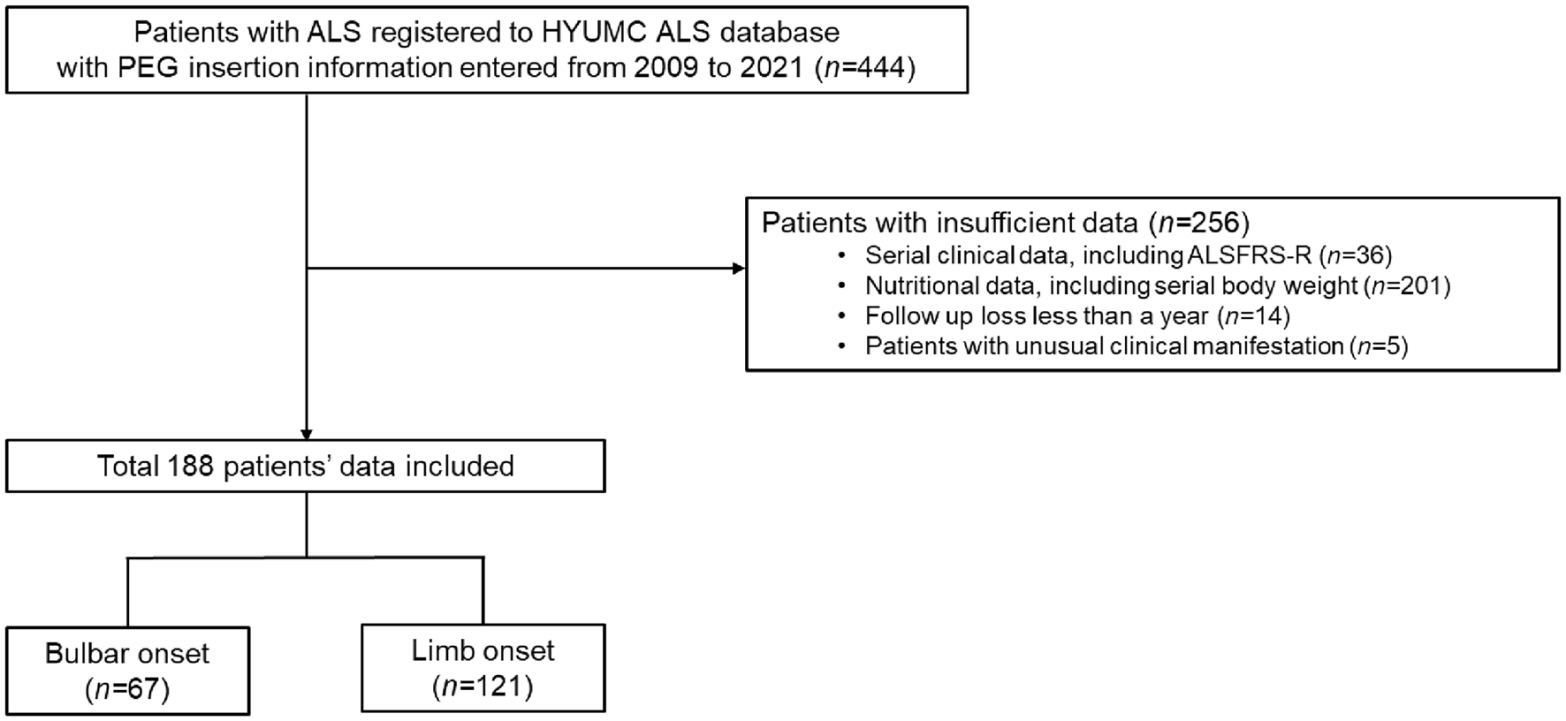
Flow chart showing the selection of study participants’ data. Data of patients with ALS who underwent PEG insertion was obtained from the ALS cohort data from Hanyang University Seoul Hospital. After excluding patients with insufficient data (n = 256), including incomplete clinical and nutritional information, loss to follow-up, and unusual clinical manifestations, we obtained a final dataset of 64 patients with bulbar onset and 121 with limb onset. ALS, amyotrophic lateral sclerosis; PEG: percutaneous endoscopic gastrostomy.

A retrospective analysis was conducted using the ALS cohort data from Hanyang University Seoul Hospital. Data of 444 patients diagnosed with definite, clinically probable, or probable laboratory-supported ALS based on the revised El Escorial criteria were reviewed^20^. Clinical and survival data related to PEG were collected from June 2009 to January 2023. Exclusion criteria were as follows: (1) lack of long-term serial data on the Amyotrophic Lateral Sclerosis Functional Rating Scale-Revised (ALSFRS-R) (n = 36); (2) lack of nutritional data, including serial body weight (n = 201); (3) loss to follow-up after PEG insertion (n = 14); and (4) presence of unusual clinical manifestations (n = 5). Ultimately, data from 188 participants were included in the analysis (Fig. 1).

### Identifying key time points for clinical data collection

To ensure a comprehensive analysis of data, including clinical profiles, ALSFRS-R scores, PEG tube insertion timing, and survival, we collected data at five critical time points as follows: (1) at diagnosis (t_1_), (2) at the time of 10% weight loss compared to the weight at diagnosis (t_2_), (3) the “recommended time point for gastrostomy(t_3_)” as the earlier time point between a 10% weight loss and demonstrated advanced dysphagia, indicated by the ALSFRS-R swallowing subscore of 2 or less, (4) at the time of PEG tube insertion (t_4_), and (5) one year after PEG tube insertion (t_5_).

We proposed “recommended time point for gastrostomy” (T_-rec_ for gastrostomy, t_3_) in this article as the optimal time point for gastrostomy. This time is marked by one of two conditions: when the patient experienced a weight loss of more than 10% compared to the weight at diagnosis, or when they show a subscore of 2 or less on question #3 (swallowing) of the ALSFRS-R, indicative of more advanced dysphagia^12,21^. The post-PEG follow-up was set at 1 year, based on a previous large cohort study where the overall mean survival after gastrostomy was 325 days, with a 95% confidence interval (CI) of 289–361 days^9^.

### Data collection

Demographic and serial clinical data, including ALSFRS-R score (ranging from 0 to 48) and body weight, were retrospectively collected from the Hanyang ALS Clinic’s ALS cohort database^22^. Changes in disease progression were calculated as the rate of decline in the ALSFRS-R total score per month between each identified time point, using the formula: difference in ALSFRS-R score between the time points of interest/duration between the time points of interest in month. Information on therapeutic agents, including US Food and Drug Administration-approved medications, such as riluzole, edaravone, and Nuedexta, was reviewed. Event data, including aspiration pneumonia, tracheostomy, and death, were collected. Participants who were alive or lost to follow-up were censored at the end of January 2023 or the last visit, respectively.

### Anthropometry

Anthropometric assessment of the participants included measurements of height, weight, body mass index (BMI), and weight loss. Height was measured once during the initial hospital visit, and weight was measured every time the patient visited the hospital for medical treatment. BMI was calculated using the following formula: weight (kg)/height × height (m^2^). To analyze the evolution in anthropometry in relation to weight loss between diagnosis and a specific point in time, the percentage of weight loss (%WL) was calculated using the formula: ([diagnostic body weight {DBW}–point of time body weight/DBW) × 100.

### Statistical analyses

Statistical analyses were performed using SAS version 9.4 (SAS Institute Inc., Cary, North Carolina, USA), and statistical significance was considered at a* p*-value < 0.05. Descriptive statistics were presented as follows: categorical variables were expressed as numbers and percentages (%), and continuous variables such as weight and BMI were expressed as mean ± standard deviations (M ± SDs). To account for the heterogeneity and variability of disease progression within our study population and to minimize the potential influence of outliers, we also included the median and interquartile range (IQR) for variables such as the ALSFRS-R score and the monthly rate of decline in ALSFRS-R score. We used different statistical analyses based on the data distribution to compare the two groups, bulbar onset, and limb onset. T-test or chi-square tests were used for normally distributed data, while the Mann–Whitney U test was used for non-normally distributed data.

Univariate and multivariate Cox proportional hazards models were utilized to calculate the hazard ratios (HRs) and 95% CIs to analyze the impact of multiple variables on survival, including age, difference in the rate of decline in the ALSFRS-R total score, BMI, weight loss, and time from symptom onset to PEG. Multiple linear regression analysis was performed to identify the characteristics associated with participants who underwent early PEG tube insertion. Graphical illustration of the ALSFRS-R score was generated using GraphPad Prism version 9.5.1 for Windows (GraphPad Software, San Diego, CA, USA).

### Ethical approval

This retrospective study was conducted in accordance with the principles outlined in the Declaration of Helsinki and received approval from the Institutional Review Board of Hanyang University (HYI-14–08-07). Written informed consent was obtained from all participants.

## Results

### Demographics and clinical characteristics of patients who underwent PEG

Table 1 presents the demographic and clinical characteristics of the study participants. In total, 188 participants were included in this study, comprising 67 (35.6%) with bulbar onset and 121 (64.4%) with limb onset. The median (IQR) age of participants at symptom onset was 57.1 (49.7–64.0) years. The median (IQR) time from symptom onset to PEG tube insertion was 30 (20–44) months, with significantly shorter intervals observed in participants with bulbar onset (23 (19–41) months) compared to those with limb onset (32 (23–47) months). During the study period, 54 (28.7%) participants experienced at least one incidence of aspiration pneumonia, and 10 (5.3%) experienced aspiration pneumonia prior to PEG tube insertion. Tracheostomy was performed in 84 participants (44.7%), with 27 (14.4%) undergoing the procedure prior to PEG tube insertion. Most participants (76.6%) received riluzole as a pharmacological treatment, while 20.7% received edaravone, and 3.7% were prescribed Nuedexta.

**Table 1:** Characteristics of the study participants (n = 188) and comparison between ALS bulbar onset (n = 67) and ALS limb onset (n = 121)^a^.

Variables	Total (n = 188)	ALS bulbar onset (n = 67)	ALS limb onset (n = 121)	*p*-value
Sex, n (%)				0.982
Male	90 (47.9)	32 (47.8)	32 (47.8)	
Female	98 (52.1)	35 (52.2)	63 (52.1)	
Age at symptom onset (years, median (IQR))	57.1 (49.7–64.0)	60.1 (53.8–67.7)	55.8 (47.5–61.0)	< 0.001
Age at PEG placement (years, median (IQR))	60.0 (53.1–66.3)	62.4 (55.9–69.5)	58.3 (51.3–63.3)	< 0.001
Site of onset, n (%)				< 0.001
Bulbar	67 (35.6)	67 (100.0)	0 (0.0)	
Limb	121 (64.4)	0 (0.0)	121 (100.0)	
Time from symptom onset to PEG (months, median (IQR))	30 (20–44)	23 (19–41)	32 (23–47)	0.012
Time from diagnosis to PEG (months, median (IQR))	19 (11–31)	13 (8–24)	23 (13–34)	< 0.001
Incidence of aspiration pneumonia, n (%)	54 (28.7)	14 (20.9)	40 (33.1)	0.078
Before PEG insertion, n (%)	10 (5.3)	2 (3.0)	8 (6.6)	0.289
After PEG insertion, n (%)	49 (26.1)	14 (20.9)	35 (28.9)	0.230
Tracheostomy, n (%)	84 (44.7)	22 (32.8)	62 (51.2)	0.015
Before PEG insertion, n (%)	27 (14.4)	5 (7.5)	22 (18.2)	0.045
After PEG insertion, n (%)	57 (30.3)	17 (25.4)	40 (33.1)	0.272
Death, n (%)	61 (32.5)	25 (37.3)	36 (29.8)	0.289
Time from symptom onset to death (months, median (IQR))	39 (28–55)	34 (24–58)	39.5 (30.3–53.3)	0.395
Pharmacological treatment options received				
Riluzole, n (%)	144 (76.6)	49 (73.1)	95 (78.5)	0.404
Edaravone, n (%)	39 (20.7)	15 (22.4)	24 (19.8)	0.679
Nuedexta, n (%)	7 (3.7)	2 (3.0)	5 (4.1)	0.691

ALS, amyotrophic lateral sclerosis; PEG, percutaneous endoscopic gastrostomy; IQR, interquartile range.

^a^Values are presented as median (IQR) or number of participants (percentage distribution), as appropriate.

### Comparison of ALS progression before and after PEG tube insertion

**Figure 2:**
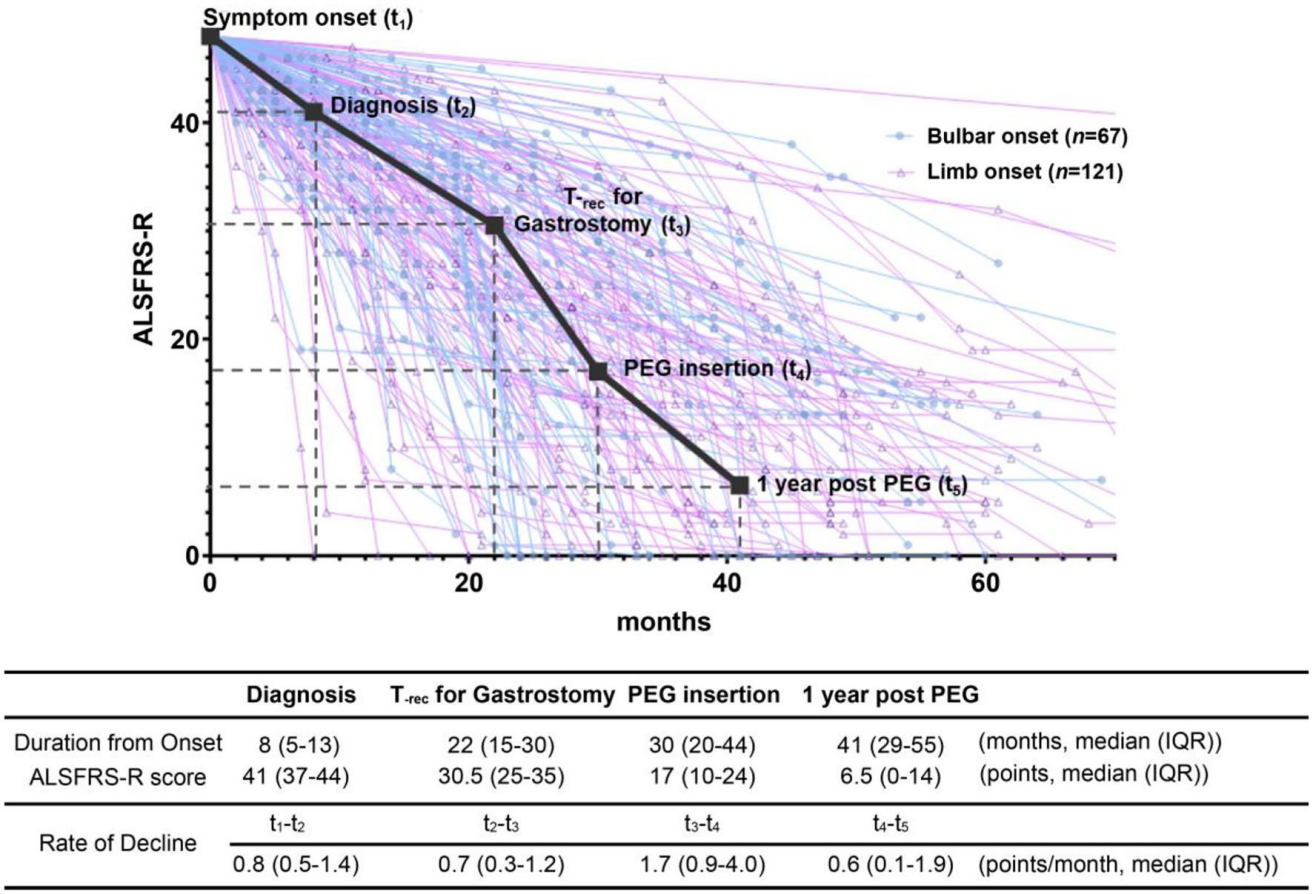
ALSFRS-R score before and after PEG tube insertion including T_-rec_ for gastrostomy. The clinical progression of patients undergoing PEG tube insertion is illustrated. The recommended time for gastrostomy (T_-rec_ for gastrostomy) was reached at a median of 22 months (IQR 15–30) after symptom onset, with the ALSFRS-R score of 30.5 (IQR 25–35) points. PEG insertion was performed 8 months after the T_-rec_ for gastrostomy, at a median of 30 (IQR 20–44) months after symptom onset, with an ALSFRS-R score of median 17 (IQR 10–24) points. The disease progression indicated by the rate of decline in ALSFRS-R score was the highest during this delay at 1.7 (IQR 0.9–4.0) points/month. After 1 year of PEG insertion, the ALSFRS-R score decreased to 6.5 (IQR 0–14) points, and the rate of decline was observed to be slower at 0.6 (IQR 0.1–1.9) points/month. ALS, amyotrophic lateral sclerosis; PEG, percutaneous endoscopic gastrostomy; ALSFRS-R, amyotrophic lateral sclerosis functional rating scale revised; T_-rec_ for gastrostomy, recommended time for PEG insertion; IQR, interquartile range.

Table 2 and Fig. 2 illustrate the progression of ALS in the context of PEG tube insertion. At the time of diagnosis, the median (IQR) interval from symptom onset to ALS diagnosis was 8 (5–13) months, and the ALSFRS-R score was 41 (37–44). The rate of decline in the ALSFRS-R score from symptom onset (t_1_) to diagnosis (t_2_) was 0.8 (0.5–1.4) points per month. The average BMI at diagnosis was 22.7 ± 3.20 kg/m^2^, indicating a risk of early malnutrition in patients with ALS during the early stages of the disease.

**Table 2:** Comprehensive flow of ALS progression before and after PEG tube insertion.

	Diagnosis(t_2_)	T_-rec_ for gastrostomy(t_3_)	PEG insertion(t_4_)	One-year post-PEG(t_5_)
Duration from onset (months, median (IQR))	8 (5–13)	22 (15–30)	30 (20–44)	41 (29–55)
Bulbar onset	9 (6–15)	20 (12–30)	23 (19–41)	34 (26–53)
Limb onset	8 (5–12)	23 (15–31.5)	32 (23–47)	43 (33.5–57)
ALSFRS-R score (points, median (IQR))	41 (37–44)	30.5 (25–35)	17 (10–24)	6.5 (0–14)
Bulbar onset	41 (37–44)	35 (30–38)	24 (16–30)	8 (0–18)
Limb onset	41 (37–44)	28 (23–34)	14 (8–19)	5 (0–13)
ALSFRS-R bulbar score (points, median (IQR))^a^	10 (9–12)	8 (6–9)	4 (3–6)	
Bulbar onset	9 (7–10)	7 (5–7)	4 (2–5)	
Limb onset	11 (10–12)	8 (7–9)	5 (3–6)	
ALSFRS-R swallowing score (points, median (IQR))^b^	3 (3–4)	2 (2–3)	1 (0–1)	
Bulbar onset	3 (3–3)	2 (2–2)	1 (0–2)	
Limb onset	4 (3–4)	2 (2–3)	1 (0–1)	
Rate of ALSFRS-R decline (points, median (IQR))^c^	0.8 (0.5–1.4)^d^	0.7 (0.3–1.2)^e^	1.7 (0.9–4.0)^f^	0.6 (0.1–1.9)^g^
Bulbar onset	0.7 (0.4–1.3)	0.5 (0.1–1.0)	2.1 (0.9–4.7)	1.0 (0.3–2.5)
Limb onset	0.8 (0.5–1.4)	0.8 (0.5–1.2)	1.6 (0.9–3.4)	0.5 (0.1–1.3)
BMI (kg/m^2^, mean ± SD)	22.7 ± 3.2	21.3 ± 3.1	19.5 ± 3.4	
Bulbar onset	22.2 ± 3.2	21.1 ± 3.3	19.7 ± 3.5	
Limb onset	23.0 ± 3.2	21.5 ± 3.0	19.4 ± 3.3	
Weight loss (%, mean ± SD)		6.1 ± 5.3	13.7 ± 11.8	
Bulbar onset		4.9 ± 5.5	11.4 ± 10.2	
Limb onset		6.7 ± 5.0	15.0 ± 12.5	

T_-rec_ for gastrostomy, Recommended time for PEG insertion; PEG, percutaneous endoscopic gastrostomy; ALSFRS-R, amyotrophic lateral sclerosis functional rating scale-revised; IQR, interquartile range; BMI, body mass index; SD, standard deviation.

^a^Sum of the bulbar subset of ALSFRS-R questions 1–3 (speech, salivation, swallowing) ranges from 0 to 12 points; ^b^Score for ALSFRS-R question 3 (swallowing) ranges from 0 to 4 points; ^c^The disease progression was measured by the rate of decline in ALSFRS-R score using the following formula: the rate of decline = (difference of ALSFRS-R score between the two-time points) / (duration between the two time points in months). ^d^The median (IQR) rate of decline from symptom onset(t_1_) to diagnosis(t_2_); ^e^The median (IQR) rate of decline from diagnosis(t_2_) to T_-rec_ for gastrostomy(t_3_); ^f^The median (IQR) rate of decline from T_-rec_ for gastrostomy(t_3_) to PEG insertion(t_4_); ^g^The median (IQR) rate of decline from PEG insertion(t_4_) to one year past PEG insertion (t_5_).

The T_-rec_ for gastrostomy was reached at 22 (15–30) months after symptom onset and 14 months after diagnosis. The median (IQR) ALSFRS-R score measured at T_-rec_ for gastrostomy was 30.5 (25–35). Participants with bulbar onset had lower bulbar scores than those with limb onset, whereas the limb-onset group showed higher weight loss at T_-rec_ for gastrostomy compared to that in the bulbar-onset group.

However, the median (IQR) time for PEG tube insertion was 30 (20–44) months after symptom onset, and 22 months after diagnosis. Compared to the median T_-rec_ for gastrostomy, the PEG insertion was delayed by 8 months following the recommendation. The median (IQR) total score of ALSFRS-R and the sum of bulbar subscores at the time of PEG insertion were 17 (10–24) and 4 (3–6) points respectively, indicating a severe decline in bulbar function. Furthermore, during the delayed time for gastrostomy, the patients showed the worst nutritional parameters, including BMI (19.5 ± 3.4 kg/m^2^) and weight loss (13.7 ± 11.8%).

In the present study, we observed a much faster rate of decline in the ALSFRS-R total score at 1.7 (0.9–4.0) points per month from T_-rec_ for gastrostomy (t_3_) to the time of actual insertion of the PEG tube (t_4_) than that from symptom onset (t_1_) to diagnosis (t_2_) being 0.8 (0.5–1.4) points per month. This finding suggests that delaying gastrostomy tube insertion can increase the rate of disease progression. However, 1 year after PEG tube insertion (t_5_), the mean ALSFRS-R score decreased by 9.2 points from 17 (10–24) to 6.5 (0–14). The rate of decline in the ALSFRS-R score from PEG tube insertion (t_4_) to 1 year after the PEG tube insertion (t_5_) decreased to 0.6 (0.1–1.9) points per month.

In summary, our findings indicate that delaying gastrostomy tube insertion in patients with significant weight loss and severe dysphagia may worsen disease progression.

### Impact of PEG on survival of patients with ALS

Survival analysis using Cox proportional hazard models revealed that the probability of survival from symptom onset to follow-up was significantly associated with several variables (Table 3). First, the initial rate of decline in the ALSFRS-R total score from the symptom onset (t_1_) to diagnosis (t_2_) showed an independent relationship with survival (HR 1.52; [95% CI 1.14–2.02]; *p* = 0.004). Second, the incidence of aspiration pneumonia before PEG tube insertion was also significantly associated with survival (HR 3.51; [95% CI 1.21–10.23]; *p* = 0.021). However, there was no significant association between survival and the time from symptom onset to diagnosis or PEG tube insertion, ALSFRS-R score, BMI, or the incidence of tracheostomy before PEG tube insertion.

**Table 3:** Impact of PEG on survival in patients with an indication for PEG (univariate and multivariate analysis with Cox proportional hazard model, n = 188).

Variables	Univariate				Multivariate			
	HR	95% CI		*p*	HR	95% CI		*p*
Bulbar onset	1.426	0.855	2.377	0.174	1.110	0.556	2.215	0.768
Time from symptom onset to diagnosis	0.972	0.941	1.004	0.084	1.012	0.962	1.065	0.646
Time from symptom onset to PEG placement	0.965	0.947	0.982	< 0.001	0.974	0.937	1.011	0.169
BMI at PEG	1.100	1.024	1.181	0.009	1.062	0.977	1.155	0.155
ALSFRS-R at diagnosis^a^	0.997	0.953	1.042	0.880	1.014	0.932	1.103	0.741
Bulbar subscore^b^	0.924	0.831	1.027	0.144	1.114	0.819	1.514	0.492
Swallowing score^c^	0.724	0.516	1.016	0.062	0.759	0.379	1.519	0.436
ALSFRS-R at PEG^a^	1.041	1.013	1.069	0.003	1.017	0.967	1.069	0.520
Bulbar score^b^	1.052	0.938	1.179	0.385	0.969	0.774	1.214	0.786
Swallowing score^c^	1.168	0.865	1.576	0.310	0.927	0.591	1.453	0.741
Rate of decline								
From onset to diagnosis^d^	1.426	1.176	1.730	< 0.001	1.523	1.148	2.020	0.004
From diagnosis to PEG^e^	1.109	0.925	1.331	0.264	0.921	0.688	1.232	0.579
From T-_rec_ to PEG^f^	1.026	0.964	1.092	0.413	0.989	0.899	1.088	0.819
Time from T_-rec_ to PEG	0.943	0.905	0.982	0.005	0.974	0.917	1.034	0.383
Incidence of aspiration pneumonia before PEG	1.733	0.693	4.335	0.240	3.513	1.206	10.229	0.021
Incidence of tracheostomy before PEG	0.582	0.250	1.353	0.209	0.503	0.182	1.388	0.185
Pharmacological treatment options received								
Riluzole	1.022	0.553	1.888	0.945	1.683	0.817	3.467	0.158
Edaravone	1.165	0.616	2.204	0.638	0.827	0.396	1.727	0.612
Nuedexta	1.379	0.334	5.694	0.657	1.053	0.219	5.054	0.949

HR, hazard ratio; CI, confidence interval at 95%; p, probability; PEG: percutaneous endoscopic gastrostomy; BMI, body mass index; ALSFRS-R, amyotrophic lateral sclerosis functional rating scale revised; ΔFS, disease progression rate; T-rec for gastrostomy, recommended time for PEG insertion.

^a^ALSFRS-R total score ranges from 0 to 48 points; ^b^bulbar subset for the sum of ALSFRS-R question 1–3 (speech, salivation, swallowing) ranges from 0 to 12 points; ^c^Score for ALSFRS-R question 3 (swallowing) ranges from 0 to 4 points; ^d^ Rate of decline from onset to diagnosis was calculated using the following formula: Rate of decline = (48—ALSFRS-R score at the time of diagnosis)/duration from symptom onset to the time of diagnosis (months). ^e^Rate of decline from diagnosis to PEG was calculated using the following formula: Rate of decline = (ALSFRS-R score at the time of diagnosis—ALSFRS-R score at the time of PEG)/duration from diagnosis to the time of PEG (months). ^f^Rate of decline from T-rec for gastrostomy to PEG was calculated using the following formula: Rate of decline = (ALSFRS-R score at the T-rec for tastrostomy—ALSFRS-R score at the time of PEG)/duration from T-rec for gastrostomy to the time of the PEG (months).

### Factors associated with early PEG tube insertion

To further understand the characteristics of patients who underwent early PEG tube insertion from our real-world retrospective data, we conducted a linear regression analysis to identify the factors associated with a shorter duration from onset to PEG tube insertion (Table 4). This analysis revealed that older age (β = − 0.34, *p* < 0.001), bulbar onset (β = − 0.13,* p* = 0.011), shorter time from symptom onset to diagnosis (β = 0.60, *p* < 0.001), lower ALSFRS-R score at diagnosis (β = 0.14, *p* = 0.032), and faster initial rate of decline from symptom onset to diagnosis to poor swallowing score (β = − 0.19, *p* = 0.002) were significantly associated with a shorter duration from diagnosis to PEG tube insertion.

**Table 4:** Factors associated with early PEG insertion in multiple linear regression analysis (n = 188).

	B	SE	β	t	*p*	VIF
Age at diagnosis	− 0.681	0.107	− 0.337	− 6.381	< 0.001	1.226
Bulbar Onset	− 5.795	2.243	− 0.134	− 2.584	0.011	1.181
Time from symptom onset to diagnosis	1.229	0.134	0.603	9.216	< .001	1.886
BMI at diagnosis	− 0.125	0.322	− 0.019	− 0.387	0.699	1.084
ALSFRS-R at diagnosis	0.535	0.248	0.138	2.157	0.032	1.787
Weight loss at severe dysphagia	0.189	0.109	0.093	1.730	0.085	1.281
Rate of decline						
From onset to diagnosis^a^	0.178	1.293	0.009	0.138	0.891	1.859
From diagnosis to weight loss 10%^b^	− 1.924	1.102	− 0.109	− 1.746	0.083	1.726
From diagnosis to swallowing F2^c^	− 3.915	1.234	− 0.199	− 3.171	0.002	1.733

B, unstandardized regression coefficient; SE, standard error; β, standardized regression coefficient; VIF, variance inflation factor, PEG, percutaneous endoscopic gastrostomy; BMI, body mass index; ALSFRS-R, amyotrophic lateral sclerosis functional rating scale-revised; swallowing F2:the timepoint when the patients get 2 points on ALSFRS-R question 3.

^a^Rate of decline from onset to diagnosis was calculated using the following formula: Rate of decline = (48—ALSFRS-R score at the time of diagnosis)/duration from symptom onset to the time of diagnosis (months). ^b^Rate of decline from diagnosis to weight loss 10% was calculated using the following formula: Rate of decline = (ALSFRS-R score at the time of diagnosis—ALSFRS-R score at the time of 10% weight loss)/duration from diagnosis to the time of the 10% weight loss (months). ^c^Rate of decline from diagnosis to swallowing F2 was calculated using the following formula: Rate of decline = (ALSFRS-R score at the time of swallowing F2—ALSFRS-R score at the time of PEG)/duration from swallowing F2 to the time of PEG (months).

## Discussion

This study presents an in-depth analysis of PEG tube insertion practices in patients with ALS in Korea. The baseline characteristics of such patients in the present study were consistent with those observed in other studies^23,24^. The age at symptom onset was median 57.1 years, with a diagnostic delay of median 8 months. Riluzole was administered to 76.6% of the participants, while 20.7% and 3.7% of the participants were prescribed edaravone and Nuedexta, respectively. Nuedexta was recently approved its potential benefits on bulbar functions, such as speech and saliva control, as well as its pseudobulbar effect. The rate of decline in the ALSFRS-R total score from symptom onset to diagnosis was 0.81 (0.46–1.35) points per month, while death occurred in 32.5% of the study population, 39 (28–55) months after diagnosis. Notably, the percentage of participants with bulbar onset (35.6%) was higher than that shown in the natural historical ALS data (20–30%)^25^, likely reflecting the inclusion of participants who had already undergone PEG.

PEG insertion was performed 19 (11–31) months from diagnosis, with a median duration of 30 (20–44) months from symptom onset. The duration between symptom onset and PEG tube insertion varied across different studies. For example, the ProGas study, which included 345 patients with ALS, showed that the average time from diagnosis to reference for gastrostomy insertion was 16.7 months, with a mean ALSFRS-R score of 28 ± 8.5 and BMI of 23.3 ± 4.4 kg/m^2^^9^. Further, a German study enrolling 89 patients with ALS reported a mean duration of 27.3 ± 20.6 months from diagnosis, with a mean ALSFRS-R score of 26.2 ± 9.3 and BMI of 21.0 ± 3.7 kg/m^2^^26^. A Spanish study of 49 patients with ALS further showed that the average time from symptom onset to PEG tube insertion was 46.9 ± 27.3 months^27^, while a Japanese study of 44 patients reported a median duration of 19.0 (13.0–24.0) months from symptom onset and a median BMI of 19.8 (18.2–23.2) kg/m^2^^28^. In our study, participants underwent PEG insertion at a similar average time but had a lower BMI compared to that reported in previous studies.

Given the heterogeneity and nonlinear progression of ALS, determining the appropriate timing for PEG tube insertion based on the patient’s individual symptoms is crucial. Therefore, in the present study, we introduced T_-rec_ for gastrostomy, which defined the recommended time point for gastrostomy as when a patient showed a weight loss of > 10% compared to the weight at diagnosis or a swallowing subscore of bulbar function of ≤ 2 in the ALSFRS-R, indicative of more advanced dysphagia. This time point was based on recommendations from the American Academy of Neurology and the European Federation of Neurological Societies^12,21^, as well as the usual clinical practice of professional neurologists, which suggests performing PEG insertion when weight loss exceeds 10% of the baseline value, bulbar symptoms decline, and forced vital capacity (FVC) exceeds 50% of the predicted level.

Our findings showed a significant delay of 8 months between the T_-rec_ for gastrostomy (t_3_) and the actual time for PEG tube insertion (t_4_), indicating a practical delay from the ideal time. This delay was concerning, particularly considering that we observed the most rapid symptom deterioration during this period (Fig. 2). Specifically, between 22 (t_3_) and 30 months (t_4_) after the onset of symptoms, we noted a decline in ALSFRS-R scores at a rate of 1.73 (0.9–4.0) points per month. Considering that the progression rate at diagnosis in our cohort, 0.81 (0.4–1.4) points per month, is consistent with the PRO-ACT database’s rate of 1.0 ± 2.3 points per month, the accelerated progression observed during delays underscores significant disease deterioration.

Overall, our results suggest that patients tend to delay PEG tube insertion until they experience critical symptoms, resulting in the most rapid disease progression. This delay was also reflected in the ALSFRS-R score at PEG insertion. Patients had a median ALSFRS-R score of 17 (10–24), a bulbar score of 4 (3–6), and a swallowing score of 1 (0–1), indicating the inability to consume food orally and an urgent need for other methods of nutritional support. Participants also showed significant weight loss (13.7 ± 11.8%), resulting in a decrease in BMI from 22.7 ± 3.2 kg/m^2^ at diagnosis to 19.5 ± 3.4 kg/m^2^ prior to PEG insertion, further demonstrating the impact of the delay in intervention.

The concerning pattern of delay is again shown that 27% of our cohort underwent tracheostomy before PEG insertion. There has yet to be a consensus regarding the optimal timing for PEG insertion in relation to respiratory function^29^. Despite these findings, literature reviews and our analysis suggest the feasibility of PEG insertion with non-invasive ventilator use or mechanical ventilation support^30–32^. Furthermore, survival outcomes following PEG insertion exhibit minimal disparity between patient cohorts with varying degrees of respiratory function^33,34^. Thus, priority should be placed on reducing the delay, but deteriorated respiratory function should not preclude patients from undergoing PEG insertion.

Our analysis revealed another interesting finding in the limb-onset group. This group was initially expected to have a longer duration from ALS symptom onset to PEG insertion (32 (IQR 23–47) months); however, it was found that this group had a higher incidence of tracheostomy before PEG tube placement and longer delays from T-rec for gastrostomy (t_3_) to PEG insertion (t_4_). When considering previous studies reported cumulative dysphagia incidences of 44%, 64%, and 72% at 1, 2, and 3 years, respectively from PRO-ACT database^23^, and the dysphagia onset averaging 20.9 ± 15.1 months after ALS symptom onset^8^, PEG insertion was significantly delayed in the limb-onset group, despite the fact that their bulbar symptoms occurred in the later stage of disease progression. Moreover, it is essential to note from a previous study that 8% of the population did not perceive dysphagia, despite evidence of dysphagia during the fiberoptic endoscopic evaluation of swallowing^10^. Therefore, regular assessments of patients’ swallowing abilities and respiratory symptoms by a multidisciplinary team and advanced care planning are crucial to prevent potential complications.

Subsequently, to determine the effect of PEG tube insertion timing on survival, we analyzed the hazard ratio using different variables. Our results identified two significant risk factors associated with reduced survival: the rate of decline in ALSFRS-R score at diagnosis and the incidence of aspiration pneumonia prior to PEG tube insertion.

As reported in previous studies^22^, our study confirmed the significance of the rate of decline from symptom onset to diagnosis again, as an index for predicting survival. However, our study showed that PEG insertion did not modify the overall progression of ALS. The estimated survival time from symptom onset to death in patients with ALS who underwent PEG insertion in our study was median 39 (28–55) months or 44.46 ± 23.33 months in average, which was not superior to the mean survival time of 50 months according to the Korean National Health Insurance System data^24^. Previous investigations have shown conflicting results with some studies have shown improvements in survival^4,35^, whereas others have not found significant benefits^17,30^.

However, based on our observations, early PEG tube insertion could potentially play a role in reducing fatal complications such as aspiration pneumonia. We found a higher incidence of aspiration pneumonia (28.7%) than that reported in previous studies^36,37^, with 5.3% of our participants experiencing aspiration pneumonia prior to PEG tube insertion. This indicates the tendency for a significant delay in PEG tube insertion, which can potentially lead to fatal complications. Moreover, prior research has shown that the survival time after aspiration pneumonia is short (median 2 months, range 0–6 months)^36^, and preventing this complication is therefore critical.

Previous studies have also highlighted the importance of PEG from the perspective of supportive management to help maintain overall health and prevent choking or dehydration and life-threatening infections, which are major contributors to the primary cause of death^4^. Our study supports this finding and suggests that although PEG insertion may not directly impact or slow disease progression, it may provide a supportive means to increase patient outcomes, possibly through weight stabilization or preventing complications, such as pneumonia, choking, or dehydration^37,38^.

The decision to insert a PEG tube is a complex process that requires careful consideration. Previous research has suggested that patients with advanced illnesses are more likely to undergo early PEG tube insertion^10^. In the present study, we investigated the factors that influenced this decision and found that dysphagia-related factors, such as bulbar onset and a faster rate of ALSFRS-R decline from diagnosis to dysphagia, tended to result in earlier PEG tube insertion (Table 4). In contrast, factors related to body weight such as BMI, weight loss with severe dysphagia, and the rate of decline from diagnosis to 10% weight loss were not significantly associated with earlier insertion.

In our experience with ALS patients, the decision to undergo PEG tube insertion often involves complex factors, including caregiver and family considerations. Previous research also supports this observation, emphasizing the significant role of families in the decision-making process^7,39^. Patients may view gastrostomy as a means of extending life and may have concerns about loss of autonomy and caregiver burdens. The family-centric approach to decision-making, particularly in Asian cultures, underscores the importance of early involvement in discussions encompassing all stakeholders, including healthcare professionals, patients, and families. The insights from our findings could serve as invaluable data to educate patients and families on why delaying optimal PEG insertion timing should be avoided to prevent the rapid progression of the disease.

Despite these strengths, this study had several limitations. First, we only enrolled participants who had undergone PEG tube insertion, which precludes comparison with patients who met the same indication criteria but opted not to undergo the procedure. Second, although the ALSFRS-R is a widely used tool to assess ALS progression, it has inherent limitations, particularly in its sensitivity to capture changes in the advanced stages of the disease. Third, the lack of serial data on respiratory function, including serial slow vital capacity (SVC) or forced vital capacity (FVC) data and post-PEG tube insertion weight changes, constrained the scope of our study. Consequently, a well-designed prospective clinical study to determine the optimal timing for PEG tube insertion and its subsequent prognosis warrants further investigation.

The clinical significance of our study lies in its demonstration of the importance of optimal PEG timing and the rationale for why PEG should not be delayed. In the course of ALS progression, timely and optimally performed PEG could mitigate the rapid deterioration associated with delayed procedure.

## Conclusions

This study provided valuable insights into using PEG in Korean patients with ALS, highlighting a median delay of 8 months from recommended to actual insertion time. Disease progression, measured by the rate of decline in the ALSFRS-R score and the BMI, peaked during this delay, emphasizing the importance of initiating early discussions regarding PEG insertion before significant weight loss or severe dysphagia occurs. Timely PEG tube insertion could also prevent fatal complications such as aspiration pneumonia. These findings provide valuable guidance for multidisciplinary teams to make informed decisions regarding PEG tube insertion and optimize patient outcomes.

## Data Availability

The datasets generated during and/or analyzed during the current study are available from the corresponding author on reasonable request.
